# Supplementary material for: MetaRibo-Seq measures translation in microbiomes
Source: Nat Commun. 2020 Jun 29;11:3268. doi: 10.1038/s41467-020-17081-z (PMC7324362; doi:10.1038/s41467-020-17081-z)
Supplement: Supplementary file 10 — Supplementary Data 7 [file 41467_2020_17081_MOESM10_ESM.zip › File2/Confidence_High_Taxonomy/84854_out.krona.html]

Javascript must be enabled to view this page.

members
magnitude
magnitudeUnassigned
count
unassigned
taxon
rank

84854\_out

4


SRS013687\_contig\_number\_contig-100\_2320.142294SRS053356\_contig\_number\_27561
2

superkingdom
2
2

2
phylum
1239

class
2
186801

186802
order
2

2
species
1897049

SRS051031\_contig\_number\_29338SRS1041145\_contig\_number\_31161
